# Oscillatory network spontaneously recovers both activity and robustness after prolonged removal of neuromodulators

**DOI:** 10.3389/fncel.2023.1280575

**Published:** 2023-12-14

**Authors:** Smita More-Potdar, Jorge Golowasch

**Affiliations:** Department of Biological Sciences, New Jersey Institute of Technology, Newark, NJ, United States

**Keywords:** neuromodulation, stomatogastric, temperature, rhythmic, homeostasis, robustness

## Abstract

Robustness of neuronal activity is a property necessary for a neuronal network to withstand perturbations, which may otherwise disrupt or destroy the system. The robustness of complex systems has been shown to depend on a number of features of the system, including morphology and heterogeneity of the activity of the component neurons, size of the networks, synaptic connectivity, and neuromodulation. The activity of small networks, such as the pyloric network of the crustacean stomatogastric nervous system, appears to be robust despite some of the factors not being consistent with the expected properties of complex systems, e.g., small size and homogeneity of the synaptic connections. The activity of the pyloric network has been shown to be stable and robust in a neuromodulatory state-dependent manner. When neuromodulatory inputs are severed, activity is initially disrupted, losing both stability and robustness. Over the long term, however, stable activity homeostatically recovers without the restoration of neuromodulatory input. The question we address in this study is whether robustness can also be restored as the network reorganizes itself to compensate for the loss of neuromodulatory input and recovers the lost activity. Here, we use temperature changes as a perturbation to probe the robustness of the network’s activity. We develop a simple metric of robustness, i.e., the variances of the network phase relationships, and show that robustness is indeed restored simultaneously along with its stable network activity, indicating that, whatever the reorganization of the network entails, it is deep enough also to restore this important property.

## Introduction

The factors that determine the robustness of complex biological systems (neuronal, ecological, molecular) have been the source of much research and debate in the past half-century, some of which being the size and heterogeneity of the networks, type and strength of connections, morphology of the components, and neuromodulation (May, 1972; Achard and De Schutter, 2006; Allesina and Tang, 2012; Chen et al., 2021; Arcuschin et al., 2023; Hutt et al., 2023; Zang and Marder, 2023). Small networks seem to defy some of the requirements for the expression of robust activity (Goldman et al., 2001; Tang et al., 2012; Doloc-Mihu and Calabrese, 2016; Haddad and Marder, 2018) due to their small size, and strong and homogeneous synaptic types, for example (Marder and Calabrese, 1996; Marder and Bucher, 2007).

Animals maintain robust behaviors such as heartbeat, respiration, locomotion, and digestion throughout their lives while facing ongoing challenges imposed by their external environment (Tang et al., 2010; Dethier et al., 2015; Doloc-Mihu and Calabrese, 2016; Ratliff et al., 2021; Arcuschin et al., 2023; Hutt et al., 2023; Zang and Marder, 2023). A global challenge all living beings currently face is changes in weather patterns and large fluctuations in local temperatures. Most proteins and enzymes are differentially regulated by temperature (Toni et al., 2019), and changes in the activity or conformation of these proteins may induce behavioral changes in animals (Angiulli et al., 2020). Poikilotherms do not regulate their body temperature and are thus particularly sensitive to body temperature changes, such as is the case of marine crustaceans, which are subject to increasingly drastic seasonal and daily ocean water temperature and pH changes. The properties of synapses and ion channels in neurons of the crab *C. borealis* are temperature sensitive (Tang et al., 2010; DeMaegd and Stein, 2020). Under such variable environmental conditions, what happens when a healthy individual suffers an injury or catches a disease? The challenge for that individual is not just to recover functional activity from the injury but also to recover the ability to withstand environmental perturbations after a certain recovery period. In mammalian (e.g., human) spinal cord injury, substantial recovery of locomotory function can be observed depending on the level of injury and age of the subject (Tillakaratne et al., 2010; Rossignol and Frigon, 2011; Pizzolato et al., 2021). However, it is not known whether the robustness, an ability to withstand external perturbations, is also restored along with recovery of the locomotion, e. g., obstacles (van Hedel et al., 2005; Fox et al., 2017). In the present study, we assessed the ability of a well-known rhythm-generating network, the *Cancer borealis* pyloric network, to homeostatically recover the robustness of its neural activity to external perturbations after the activity has been severely disrupted. We disrupted activity by experimentally removing neuromodulatory inputs (i.e., decentralization), and we used temperature changes as an external perturbation to probe and evaluate activity robustness during the period the system is recovering its activity. It is interesting to note that spinal cord injury and decentralization experiments have in common the fact that neuromodulatory inputs, often released by so-called command neurons to local central pattern generators are lost, and several studies have shown functional recovery of rhythmic motor activities after removing important command signals (Barbeau and Rossignol, 1987; Luther et al., 2003; Sakurai and Katz, 2009; Herman et al., 2018; Golowasch, 2019). In the pyloric network of the crab, *C. borealis*, activity that is nearly indistinguishable from that observed before decentralization recovers in one to a few days (Luther et al., 2003). Here, we address whether the recovered activity is also robust to external perturbations it typically encounters.

The pyloric network of the stomatogastric ganglion (STG) of *C. borealis* generates an ongoing rhythm (∼1 Hz) with a complex patterned movement of the animal’s pylorus, which is thought to filter food (McGaw and Curtis, 2013). It has been shown that the pyloric network generates robust activity, with the network neurons maintaining stable phase relationships over a wide temperature range (7–27°C) when its neuromodulatory inputs are intact (Tang et al., 2010; Soofi et al., 2014; Haddad and Marder, 2018). However, the experimental removal of neuromodulators (decentralization) immediately alters both the pyloric cycle frequency and the activity phases of motor neurons (Luther et al., 2003) and makes the rhythm less robust to temperature changes (Haddad and Marder, 2018). Supplying neuromodulators exogenously stabilizes the rhythm and brings the robustness to temperature changes back to control levels (Haddad and Marder, 2018). As mentioned earlier, 24 h after decentralization, the pyloric network can recover a functional rhythm, albeit at a frequency somewhat lower than control. We know that neuromodulatory inputs, however, are not restored (Luther et al., 2003). We hypothesize that the robustness of the pyloric rhythm is also restored as its activity recovers after decentralization.

**FIGURE 1:**
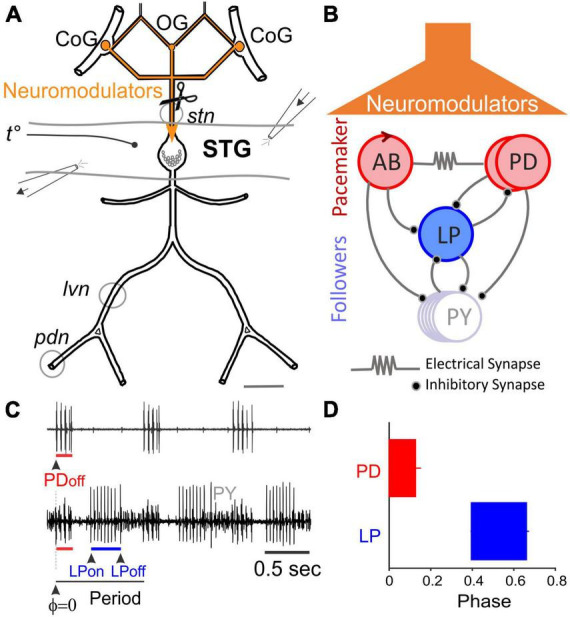
The Stomatogastric Nervous System (STNS). **(A)** Schematic diagram of STNS nerves and ganglia. The paired commissural ganglia (CoG) and the esophageal ganglion (OG) contain many neuromodulator-releasing neurons that project and release their modulators onto the stomatogastric ganglion (STG) via the stomatogastric nerve (*stn*). The axons of pyloric motor neurons run posteriorly through the lateral ventricular nerve (*lvn*), pyloric dilator nerve (*pdn*), and other nerves. Gray circles around *lvn* and *pdn* represent petroleum jelly wells built to record extracellular pyloric activity. Perfusion of the STG was performed using an open-ended well inside of which temperature was recorded. A small well around the *stn* was used to add TTX and decentralize the STG. **(B)** Simplified diagram of the pyloric network. The pacemaker interneuron, Anterior Burster (AB), is coupled to two Pyloric Dilator neurons (PD) via gap junctions and together form the pacemaker unit. Lateral Pyloric (LP) and Pyloric (PY) neurons are follower motor neurons. All chemical synapses are inhibitory. **(C)** Extracellular motor nerve recordings showing rhythmic activity. Phase is the relative timing of the onset or end of a neuron’s activity within a cycle period. **(D)** Conventionally, the first spike of the PD burst is the PD_*on*_ phase and also the reference point to measure other pyloric phases (PD_*off*_, LP_*on*_, and LP_*off*_).

Here, we define robustness as the ability of a system to maintain functional properties when subjected to a range of external perturbations. An important property of the pyloric activity pattern is the activity phase relationships of the different neurons in the network (Marder and Bucher, 2007; Soofi et al., 2014). Thus, we quantified robustness as the statistical variance of several phase relationships (Figures 1C, D) across the perturbation range. We used brief temperature changes as the external perturbation to probe the system’s robustness. We measured pyloric phase relationships at a wide range of temperatures at four time points within the first 24 h after decentralization. We compared the variances across the temperature range at these different time points and found that the variance of each phase across temperatures increases relative to control shortly after the first few hours of decentralization. However, after 24 h, a marked decrease in variance back toward control values is observed. This trend in phase variances indicates that the temperature robustness of pyloric activity significantly drops immediately after the removal of neuromodulators but spontaneously begins to recover several hours later.

## Materials and methods

### Animals

Male Jonah crabs, *Cancer borealis*, were purchased from a local seafood market (Seabras, Newark, NJ, USA). They were housed in our animal tanks at around 11–12°C in circulating, aerated artificial ocean water (InstantOcean, Blacksburg, VA, USA). The data presented in this study were collected between September 2021 and December 2022.

### Physiological saline

*Cancer borealis* saline contained 440 mM NaCl, 26 mM MgCl_2_, 13 mM CaCl_2_, 11 mM KCl, 10 mM Tris base, and 5 mM maleic acid, buffered to pH 7.4–7.5.

### Dissection and desheathing

Male crabs were anesthetized by placing them in ice for approximately 30 min. The stomatogastric nervous system (STNS, Figure 1A), situated dorsally on the crab’s stomach, was dissected as previously described (Gutierrez and Grashow, 2009) and pinned dorsal side up in a Sylgard-lined Petri dish. Most of the times two preparations were placed in the same dish to increase the rate of data acquisition.

The STNS consists of four ganglia: a pair of commissural ganglia (CoGs), the esophageal ganglion (OG), and the stomatogastric ganglion (STG) (Figure 1A). The sheath over the dorsal side of the STG was excised with fine tungsten pins. The STG in *C. borealis* contains 26 neurons that form two rhythmic pattern-generating networks: the pyloric and gastric networks. The CoGs and the OG contain the cell bodies of modulatory neurons whose axons run caudally through the stomatogastric nerve (*stn*) and release a variety of neuromodulators in the STG neuropil (Figure 1A). In the presence of neuromodulators, the pyloric network (Figure 1B) generates a triphasic rhythm with a cycle frequency of ∼1 Hz (Figure 1C).

### Decentralization

Decentralization is the experimental isolation of the STG from neuromodulatory inputs. To decentralize, a petroleum jelly well was first built around the *stn* (Figure 1A). It was then filled with saline containing 10^–6^ M tetrodotoxin (TTX, Alomone Labs). TTX blocks action potential transmission along axons of neuromodulatory projection neurons and, thus, blocks the release of neuromodulators in the STG neuropil. Subsequently, the *stn* was transected inside the well. TTX in the well prevents injury discharges and possible neuromodulator release during transection. Transection was included to prevent possible resumption of action potential conduction if TTX-free saline accidentally enters the well during the 24 h incubation period.

### Electrophysiological recordings

The pyloric rhythm was recorded extracellularly with stainless steel pin electrodes placed inside petroleum jelly wells built around the lateral ventricular nerve (*lvn*) and the pyloric dilator nerve (*pdn*), as shown in Figure 1A. Among others, the *lvn* carries LP, PY, and PD motor neuron axons, whereas *pdn* carries axons of PD neurons only. Extracellular electrodes were connected to a differential AC amplifier (Model 1700, AM Systems). The STG was enclosed in an open-ended elongated petroleum jelly chamber and perfused constantly with recirculating saline through a large 2-liter reservoir for the duration of the experiments (≥24 h). The saline temperature was maintained at ∼12°C with a homemade Peltier cooling system, except when temperature changes were performed. The pyloric rhythm and STG temperature were recorded continuously. The ends of the petroleum jelly well around the STG (Figure 1A) were kept open to ensure that the nerves and the rest of the nervous system were also minimally perfused even if the temperature was different from that at the STG. For the entire duration of the experiment, the STNS preparations were placed on the electrophysiology setup and perfused in saline containing no antibiotics. The volume of the Petri dish was approximately 20 ml.

### Temperature changes

As indicated before, we used temperature changes as a means to perturb the pyloric system and probe its robustness during its spontaneous recovery from decentralization. We assume that these temperature changes do not significantly impact the process of recovery itself, although we acknowledge that they may delay or advance it. We did not examine these possibilities in this study. We performed these tests at four time points: 0 (before decentralization), and 0.5, 6, and 24 h after decentralization. The temperature of the perfusion saline was modified by manually adjusting the power to the homemade Peltier device. A digital thermometer (Warner TC-344 B) was placed in the elongated Vaseline chamber and within a few millimeters of the STG (Figure 1A). At each time point the temperature was set to 9, 12, 15, 18, 21, 24, 27, and 30°C, in that order. Each step took 2–3 min. When the temperature had reached a stable level (within ± 0.3°C), activity was recorded for 120 s. Each series took approximately 45 min to complete.

### Data analysis and statistics

The pyloric activity was characterized by the cycle frequency and the phase relationships of the different pyloric neurons. As is customary, the first spike of the PD neuron burst recorded on the *pdn* was defined as the onset of the PD burst (PD_*on*_) and of the pyloric period. The onset and end of the bursts of other neuron types were measured relative to PD_*on*_ (Figures 1C, D). In this manner, three pyloric phases (PD_*off*_, LP_*on*_, and LP_*off*_) were determined (Figure 1D). All the pyloric cycles recorded in a 2-min window were used to calculate the corresponding pyloric frequency and phase averages, standard deviations and variances (StDev^2^). Generalized Linear Model, Bootstrapping and ANOVA were used to determine the effects of time after decentralization and of temperature on both intact (control) and decentralized preparations. The Generalized Linear Model (GLM) test was used to compare the variances calculated for each phase. GLM identifies non-linearities in the observations and transforms them into linear functions (using a specific Link function, here “Gamma”) by identifying a relationship between measured variables and, then, predicting their values by linear interpolation. Data are sometimes difficult to obtain (and thus sometimes lost) over the course of these long experiments (see sample numbers in Table 1). The GLM test is particularly useful with our data since, by transforming and linearizing the data, missing values are accurately predicted by interpolation. Both the GLM and Bootstrapping methods have the important advantage of expanding the effective datasets: by predicting the missing data after linearization of the time-dependence of the data (typical in these long-lasting experiments) in the case of GLM, and by the process of bootstrapping datasets whose distributions are not the same, thus making these distributions comparable.

**TABLE 1:** Pyloric rhythm frequencies in control and decentralized conditions at various temperatures.

Control frequencies (means ± SD)						Decentralized frequencies (means ± SD)			
Temp		0 h	0.5 h	6 h	24 h	0 h	0.5 h	6 h	24 h
9°C	Mean	1.07	1.04	0.96	0.74^a,b,c^	1.02	0.32^a^	0.34^a^	0.44^a^
	SD	0.15	0.17	0.14	0.27	0.13	0.14	0.23	0.22
12°C	Mean	1.34	1.27	1.14	0.92^a,b,c^	1.27	0.36^a^	0.33^a^	0.48^a^
	SD	0.18	0.23	0.20	0.35	0.20	0.20	0.23	0.26
15°C	Mean	1.64	1.47	1.40	1.07^a,b,c^	1.55	0.45^a^	0.34^a^	0.49^a^
	SD	0.27	0.30	0.29	0.46	0.30	0.22	0.25	0.31
18°C	Mean	1.95	1.70	1.61^a^	1.26^a,b,c^	1.83	0.61^a^	0.48^a^	0.71^a,c^
	SD	0.34	0.34	0.43	0.62	0.37	0.28	0.27	0.33
21°C	Mean	2.08	1.90	1.64^a^	1.28^a,b,c^	2.03	0.84^a^	0.70^a^	0.96^a^
	SD	0.39	0.37	0.43	0.39	0.40	0.32	0.30	0.39
24°C	Mean	2.17	2.05	1.64^a,b^	1.52^a,b^	2.08	1.15^a^	0.85^a^	1.23^a^
	SD	0.38	0.44	0.38	0.39	0.41	0.39	0.36	0.38
27°C	Mean	2.37	2.34	1.71^a,b^	1.93^a,b^	2.32	1.58^a^	1.18^a^	1.49^a^
	SD	0.54	0.47	0.42	0.59	0.42	0.43	0.42	0.48
30°C	Mean	2.88	2.83	2.43^a^	2.46^a^	2.75	2.08^a^	1.95^a^	1.99^a^
	SD	0.59	0.65	0.79	0.72	0.72	0.64	0.79	0.59
	N	22–25	11–13	20–24	14–20	26–30	15–17	19–23	15–21

Pyloric rhythm frequencies (mean ± SD) measured over ≥120 sec at each indicated temperature (left), condition (top), and time from the beginning of recordings. In control preparations neuromodulatory inputs were intact throughout 24 h. For decentralized preparations, times after initial (0 h) recordings correspond to times after decentralization. Statistical significance (superscript letters) applies within the conditions (Control or Decentralized) and was obtained from a Generalized Linear Model test.

^a^*P* < 0.05 between 0 h and indicated times.

^b^*P* < 0.05 between 0.5 h and indicated times.

^c^*P* < 0.05 between 6 and 24 h. Data were obtained from 25 preparations (Control) and 30 (Decentralized). The uneven sample sizes (bottom row) reflect the fact that at different temperatures and conditions, some preparations cease their rhythmic activity, or it could otherwise not be recorded.

Statistical analyses were performed using MATLAB R2022b, Rstudio, and SigmaStat software packages (SPSS, Chicago, IL, USA). Data are reported as means and standard deviation of the mean unless otherwise stated.

## Results

### Recovery of the pyloric rhythm

**FIGURE 2:**
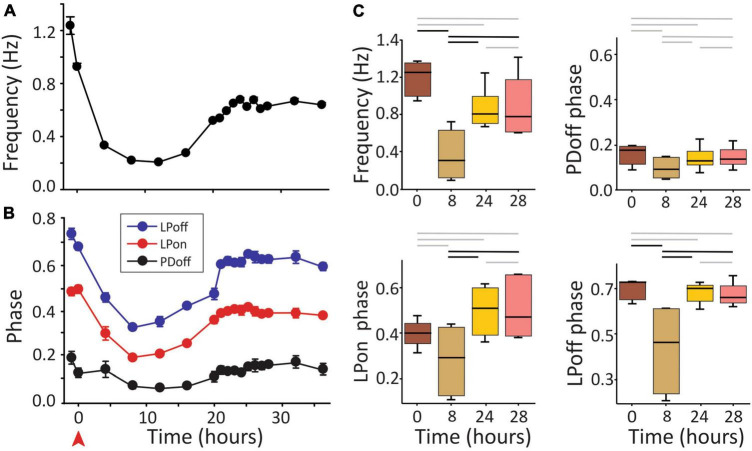
Pyloric activity and removal of neuromodulatory input. **(A)** Pyloric network frequency and **(B)** phases over the course of 36 h in response to decentralization (red arrowhead). Frequency recovers to within 75% of control **(A)** and phases to within ∼10% of control levels **(B)** after approximately 24 h in this preparation. Error bars in **(A,B)** are standard deviations of the mean of 2 min of recording (∼100 pyloric cycles). **(C)** Medians and 25, 75% quartiles at the times indicated (decentralization immediately after *t* = 0). Statistical comparisons were obtained with a Generalized Linear Model test. Black horizontal bars indicate statistical significance between the time points at the end of each bar (*P* < 0.05). Gray bars indicate a lack of statistical significance. *N* = 6 preparations.

A large decrease in pyloric frequency and phase relationships are known to occur upon the removal of neuromodulatory input to the network, i.e., after decentralization. In *C. borealis* this is followed by a complete recovery of pyloric phases and a partial recovery of the pyloric frequency within 24 h (Golowasch et al., 1999b; Luther et al., 2003), although conflicting reports of this phenomenon have been published (Hamood et al., 2015). All those experiments were conducted at a constant temperature of ∼12°C. Our first goal was to confirm the original observations of Luther et al. (2003). Thus, we first decentralized the STNS preparations and then maintained them at 12°C for at least 24 h, as done in earlier studies. During this period, the pyloric network activity was monitored continuously. Figure 2 shows an example of the pyloric activity of one preparation that we monitored for 36 h after decentralization. In this example, frequency recovered to approximately 75% of control levels (Figure 2A), and phases recovered to levels within at least 90% of the control values within 24 h. They remained stable for at least 4 h (and 12 h in this example) (Figure 2B), consistent with results of Luther et al. (2003). In Figure 2C, we show the statistical analysis on a small set of experiments (*N* = 6). All measured phases and the pyloric frequency decreased at 8 h after decentralization and recovered stably (for at least 4 h) by 24 h. PD_*off*_ decreased, but the change did not reach a significant effect at 8 h in this sample. In the context of these and previous results, we conclude that the effect of decentralization is, as reported before (Luther et al., 2003), a temporary reduction in phases and frequencies followed by their spontaneous and stable recovery (partial for frequency and nearly complete for phases) after approximately 24 h in the continuous absence of neuromodulators.

Once confirmed that the pyloric system can recover its essential functional properties (frequency and phase) at the standard 12°C as shown before, we examined the frequency and phase responses of the system to a large range of brief temperature challenges during the recovery period. To this end, we tested the robustness of the network at a range of temperatures from 9 to 30°C.

### Frequency

**FIGURE 3:**
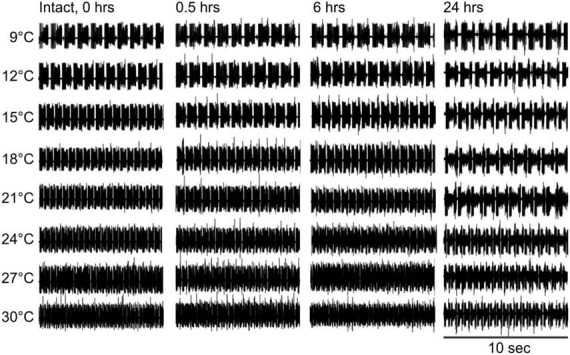
Control pyloric activity responses to temperature changes. Intact pyloric network activity recorded from the *lvn* at four different times **(top)** and at eight temperatures **(left)**. Rhythm frequency increases with temperature and slightly decreases over time at all temperatures.

**FIGURE 4:**
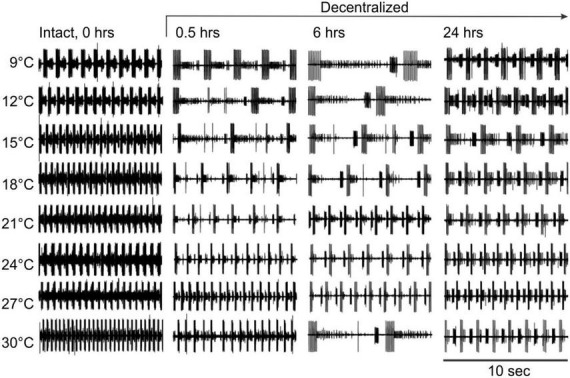
Pyloric network activity responses to temperature changes in the absence of neuromodulators. Pyloric network activity was recorded from the *lvn* at eight temperatures (left) before (Intact, 0 h) and at three different times (0.5, 6, and 24 h) after decentralization. Rhythm frequency increases with temperature. After decentralization, the overall rhythm frequency first decreases over the first few hours and then increases around 24 h after decentralization.

**FIGURE 5:**
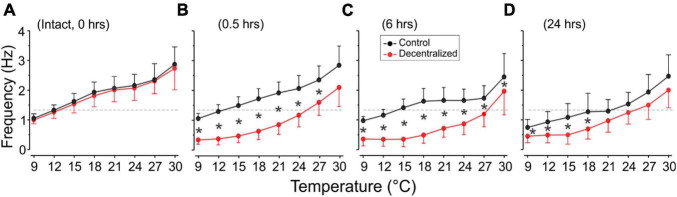
Pyloric network frequency recovery. The frequency of the pyloric network activity was measured as the inverse of the period (Figure 1) and is plotted as a function of test temperatures recorded extracellularly from the *lvn* or *pdn*. We show temperature effects in the intact preparation (**A**: 0 h) and at three different times thereafter: **(B)** 0.5, **(C)** 6, and **(D)** 24 h (top). In decentralized preparations (red), conduction along the *stn* was blocked with TTX and then by severing it (decentralization) immediately after the 0 h recordings were completed. Rhythm frequency increases with temperature in all conditions. Rhythm frequency slightly decreases in control (non-decentralized preparations) over time, and drastically decreases immediately after decentralization (0.5 h), and then starts to increase between 6 and 24 h back toward control levels. Black stars (*) indicate statistically significant differences in frequency between control (black) and decentralized (red) preparations at the corresponding temperatures. Significance values are determined with a Generalized Linear Model test (link function: Gamma). Symbols indicate *P* ≤ 0.05. Sample numbers for each point varied, as indicated in Table 1.

Under control conditions (not decentralized), increased temperature raised the cycling frequency, but phases remained stable across time—see also (Tang et al., 2010). By 24 h, however, the frequency was somewhat lower than controls (0 h) at all temperatures (Figure 3 and Table 1, left) suggesting that some run down of the preparations happens in that period. In decentralized preparations (Figure 4), the effect of temperature was similar to control: there was an increase in pyloric frequency as temperature increases (Figure 4), as reported before for intact (non-decentralized) preparations (Tang et al., 2010). However, the frequency drastically dropped immediately after decentralization at all temperatures tested (Figures 4, 5B). The frequency then climbed back to values closer to control but typically did not reach control levels (Figures 4, 5C, D and Table 1, right; Thoby-Brisson and Simmers, 1998; Luther et al., 2003).

Interestingly, the range of frequencies attained (lowest to highest) as a function of temperature (i.e., the sensitivity of the preparations to temperature-dependent frequency changes) remained virtually unchanged at all times and conditions (Figure 5): Control preparations (frequency range (maximum–minimum) at 0 h = 1.81 Hz, 0.5 h = 1.79 Hz, 6 h = 1.47 Hz, and 24 h = 1.72 Hz). A One-way ANOVA on the residuals (average-subtracted frequencies) to test if these ranges across the eight temperatures tested at each time point (0–24 h) are indeed the same, showed no difference between time points 0 and 24 h (*F* = 0.385, *P* = 0.764, df = 31, power alpha = 0.049). Likewise, among decentralized preparations (frequency range at 0 h = 1.73 Hz, 0.5 h = 1.76 Hz, 6 h = 1.62 Hz, and 24 h = 1.55 Hz), a One-way ANOVA on residuals showed no difference between time groups (*F* = 0.126, *P* = 0.944, df = 31, power alpha = 0.049). Furthermore, when comparing the combined frequency residuals for all time points under each condition, showed no statistically significant difference between Control and Decentralized preparations (*F* = 0.396, *P* = 0.532, df = 63, power alpha = 0.047, One-way ANOVA). This suggests that the sensitivity of the pyloric activity frequency to temperature changes is not affected by either the presence or absence of neuromodulators or the state of recovery from decentralization.

### Phase relationships

**FIGURE 6:**
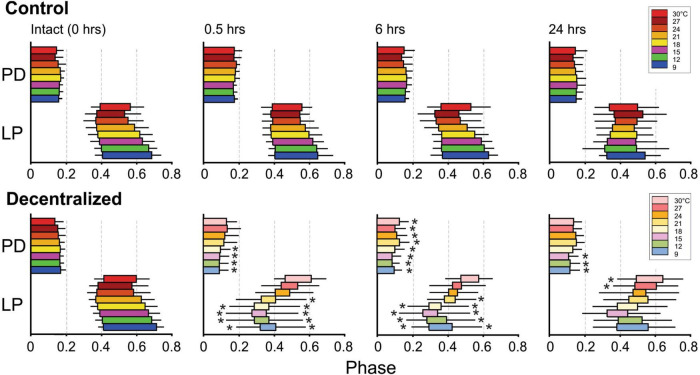
Pyloric network phase recovery. Horizontal bars represent the burst phase onset to the end of the PD and LP neurons at different times between 0 and 24 h **(top)** and at different temperatures (colors). The PD_*off*_, LP_*on*_, and LP_*off*_ phases were measured as described in Figures 1C, D. Black horizontal lines correspond to standard deviations of the PD_*off*_, LP_*on*,_ and LP_*off*_ phases (PD_*on*_ phase has mean and SD of zero by definition). Black stars (*) indicate statistically significant differences in mean phase between control **(top)** and decentralized **(bottom)** preparations at the corresponding temperatures (at *P* < 0.05). Significance values are determined with a Generalized Linear Model test (link function: Gamma). Sample numbers vary between temperatures, as described in Figure 5 and Table 1.

It was previously reported that phase relationships are significantly affected by decentralization but that they recover to levels indistinguishable from control levels within 24 h in *C. borealis* (Luther et al., 2003) and a few days in the lobster *Jasus lalandii* (Thoby-Brisson and Simmers, 1998). As mentioned, we confirmed these results for *C. borealis* at a constant temperature of 12°C throughout the experiment (Figure 2) but also expanded them to examine this recovery at a wide range of temperatures. Figure 6 shows the beginning and end phases of PD and LP neurons at eight temperatures, each at four time points, and each of these under two different conditions: Control and Decentralized. At all times in between temperature tests, the preparations were kept at 12°C. The green bars correspond to values obtained at 12°C test temperature, similar to those published before (Luther et al., 2003). Our data clearly show that all 3 phases (PD_*off*_, LP_*on*_, and LP_*off*_) are disrupted immediately after decentralization, particularly at test temperatures closest to the animals’ natural habitat temperature (9–18°C) (Figure 6 bottom, 0.5, 6 h). However, all three phase relationships within most of this temperature range recovered to values indistinguishable from time-matched controls after 24 h, surprisingly with the exception of PD_*off*_ at 9 and 15°C. The lack of a significant recovery at these temperatures is likely due to under sampling since the phases do increase in the direction of control levels, similar to what we observed at the other temperatures. The temperatures for which the system (specifically LP phases) seems to be less able to recover completely are the highest temperatures in this range (21–30°C; Figure 6). Nevertheless, the trend toward values increasingly closer to control is apparent (see section “Discussion”).

In what follows, we analyze the variance of the phases recorded across a range of eight temperatures as a measure of the robustness of the system.

### Robustness

A decrease in the robustness of stomatogastric neuronal networks to temperature variations due to short-term decentralization has been demonstrated before (Stadele et al., 2015; Haddad and Marder, 2018). In both cases the authors showed that peptide neuromodulators are crucial to maintaining the robustness of both the pyloric and gastric networks, respectively, when challenged with a range of temperature changes. As we show above, activity is spontaneously restored within a few hours (∼24 h) after removing neuromodulatory input (decentralization) to levels very close or indistinguishable from control. Our goal here was to determine whether robustness to external perturbations is also a network property that can recover spontaneously after decentralization. We used temperature changes between 9 and 30°C as a perturbation to challenge the network and determine its robustness. Consistent with our original definition, we quantified robustness as the variance of each of the three phases of the pyloric rhythm: PD_*off*_, LP_*on*_, and LP_*off*_ (see Figures 1C, D) across the temperature range. We did not consider pyloric network frequency in this determination because of its known high level of variability between animals (Luther et al., 2003; Hamood and Marder, 2014). We first assessed the changes in variability using a Generalized Linear Model test on the variances of the phase relationships. We then confirmed these results with another independent test: bootstrapping of these variances.

**FIGURE 7:**
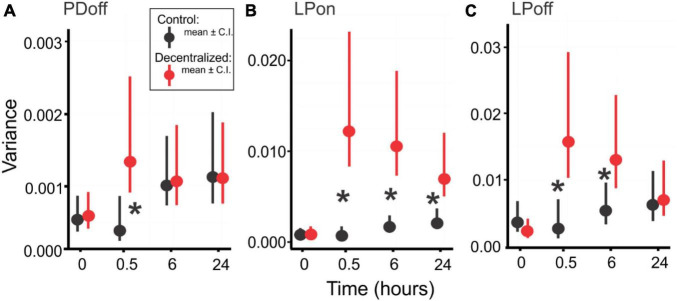
Pyloric network robustness. Variances of PD_*off*_ **(A)**, LP_*on*_ **(B)** and LP_*off*_ **(C)** phases across a 9 to 30°C temperature range at four time points, in Control (Black) and Decentralized (Red) conditions. Symbols are average variances across preparations, and vertical bars are confidence intervals. Black stars (*) indicate a significant difference (*P* < 0.05) between Control and Decentralized conditions at the respective time points. Significance values determined with a Generalized Linear Model test (link function: Gamma). Statistical details are listed in Table 2.

We used the same set of STNS preparations described before to quantify robustness as defined above. We first calculated variances of each phase across the temperature range for individual preparation. We then used a Generalized Linear Model (GLM) test to compare the variances thus calculated. Figure 7 shows the average variances at different time points and for Control and Decentralized preparations. All three phase variances significantly increased 30 min after decentralization (red symbols) and then began to decrease again toward control values (black symbols). At 24 h, the variances of two of the three phases reached values indistinguishable from control levels. The exception is the LP_*on*_ phase variance (Figure 7B), which, although lower than at the 0.5- and 6-h time points, remains statistically significantly different from control levels. To examine whether the recovery of robustness is affected by the extreme temperatures considered, we performed the same analysis on a reduced temperature range, that corresponds to the current range of temperature changes in the north Atlantic ocean: 9–18°C, as reported by the National Oceanographic and Atmospheric Administration, NOAA.^1^ With this data set, our conclusions are confirmed, and all three phases clearly show a rapid increase in variance after decentralization and full recovery to time-matched control levels (Table 3). We interpret these results to indicate that the temperature robustness of the pyloric rhythm phases decreases considerably after decentralization but is spontaneously and homeostatically restored even in the absence of neuromodulatory inputs after a recovery period of at least 24 h.

**TABLE 2:** Pyloric rhythm variances across a 9–30°C temperature range.

	Time (hours)	Coefficient estimate	SE	t ratio	*p*-value
PD_off_	0	−5.21e-05	0.000152	-0.342	0.7325
	0.5	-9.38e-04	0.000338	-2.774	**0.0062**
	6	-4.74e-05	0.000307	-0.154	0.8776
	24	1.55e-05	0.000349	0.045	0.9645
LP_on_	0	-0.000161	0.000284	-0.567	0.5713
	0.5	-0.011510	0.002960	-3.888	**0.0001**
	6	-0.008855	0.002328	-3.803	**0.0002**
	24	-0.004989	0.001585	-3.147	**0.0020**
LP_off_	0	0.00126	0.00117	1.079	0.2824
	0.5	-0.01231	0.00384	-3.210	**0.0016**
	6	-0.00725	0.00311	-2.334	**0.0208**
	24	-0.00100	0.00231	-0.434	0.6647

Pyloric rhythm phases PD_off_, LP_on_, and LP_off_ were compared between decentralized and time-matched controls at each time point with a Generalized Linear Model (GLM) test with link function Gamma. Average variances for each condition are listed. Time-dependent comparisons. The mean of GLM coefficient estimates across 9–30°C temperature range correspond to the slope of the linear trend (per log unit) generated by the GLM test during the control vs. decentralized comparison. SE is standard error of the coefficient estimate; t ratio and *P*-values correspond to the control vs. decentralized comparisons. Significant differences between control and decentralized are highlighted in bold. Df = 160.

**TABLE 3:** Pyloric rhythm variances across a 9–18°C temperature range.

	Time (hours)	Coefficient estimate	SE	t ratio	*p*-value
PD_off_	0	7.64e-06	0.000049	0.158	0.8757
	0.5	-0.00015	0.000120	-1.310	0.1978
	6	-0.00026	0.000119	-2.202	**0.0292**
	24	1.99e-05	0.000189	0.105	0.9169
LP_on_	0	-0.00016	0.000176	-0.949	0.3441
	0.5	-0.00364	0.001221	-2.979	**0.0034**
	6	-0.00340	0.001119	-3.032	**0.0029**
	24	-0.001324	0.000733	-1.805	0.0730
LP_off_	0	0.00084	0.000559	1.503	0.1350
	0.5	-0.00458	0.001817	-2.518	**0.0128**
	6	-0.00315	0.001419	-2.220	**0.0279**
	24	0.00155	0.001283	1.207	0.2293

Pyloric rhythm phases PD_off_, LP_on_, and LP_off_ were compared between decentralized and time-matched controls at each time point with a Generalized Linear Model (GLM) test with link function Gamma. Average variances for each condition are listed. The mean of GLM coefficient estimates across 9–18°C temperature range correspond to the slope of the linear trend (per log unit) generated by the GLM test during the control vs. decentralized comparison. SE is standard error of the coefficient estimate; t ratio and *P*-values correspond to the control vs. decentralized comparisons. Significant differences between control and decentralized are highlighted in bold. Df = 153.

**FIGURE 8:**
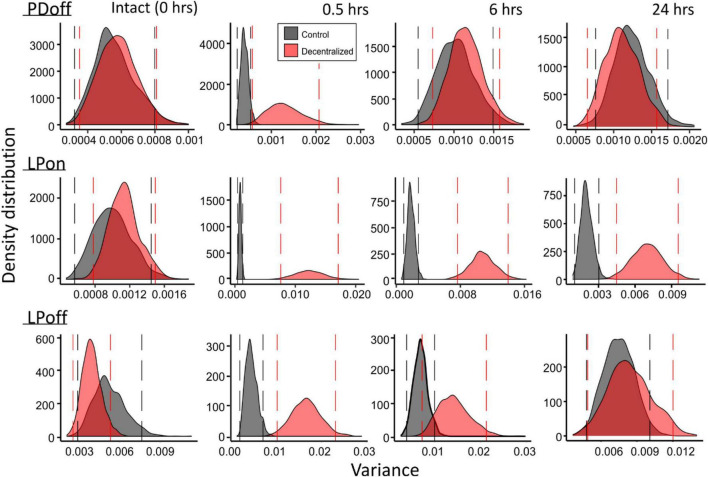
Bootstrapping of phase variances. Each of the variance data sets at the four time points (top) and two conditions (red vs. black distributions) were bootstrapped for 1000 iterations. Density distributions of bootstrapped variance across a 9 to 30°C temperature range are shown for each. Vertical lines mark 95% confidence intervals for each distribution. Separation of the distributions past the confidence intervals indicates significance of differences of the distributions.

To confirm our results, we performed a bootstrapping analysis (see section Materials and methods) on the same variances used for the GLM analysis. Figure 8 shows the distributions of 1000 bootstrapping iterations for each pyloric phase (PD_*off*_, LP_*on*_, LP_*off*_). An overlap of the Control and Decentralized distributions past the confidence intervals marked by the vertical lines indicates a lack of statistically significant differences. Thus, we observe the same pattern of changes over the course of 24 h as revealed by the GLM analysis: all measured phase variances became significantly different at time 0.5 h and then began to recover. PD_*off*_ and LP_*off*_ variances fully recovered by 24 h, and LP_*on*_ variance, although the distributions clearly moved closer to each other than they were at the 0.5 and 6 h time points, they remained significantly apart (different) at the 95% confidence level.

## Discussion

In the pyloric network of *C. borealis*, neuromodulation is essential for the network to express robust responses to temperature changes (Haddad and Marder, 2018). Removal of neuromodulatory input, i.e., decentralization, severely destabilizes and sometimes eliminates the pyloric rhythm (Thoby-Brisson and Simmers, 1998; Luther et al., 2003), as well as its robustness to external perturbations (Haddad and Marder, 2018). Remarkably, after about 24 h of the continued absence of neuromodulatory inputs, pyloric activity features regain control (or near-control) levels (Figures 2, 5, 6) as previously reported (Luther et al., 2003). Moreover, once recovered, phases and frequency remain stable for several hours (Figure 2). Here, we hypothesize that the robustness of the pyloric activity to perturbations (in the form of temperature changes) is also homeostatically restored in the prolonged absence of neuromodulators. We show that, (1) the pyloric network phase relationships recover to values close to or indistinguishable from control values as shown before (Luther et al., 2003), and (2) robustness, characterized by the variance of the phase relationships of the different neurons in the network in response to temperature changes, is also restored.

A keen observer may argue that the control variance values shown in Figure 7 are themselves not stable and rise over time, suggesting that the robustness of the control preparations is weakening. Two things are important in this regard. First, the changes are not statistically significant (Table 4). Thus, while a small rise in variance is visible, indicating that the preparation, after several hours in minimal culture conditions, does somewhat deteriorate or run down (as assessed by slight color and tissue transparency changes, as well as the fragility of neurons upon impalement), the rise is not statistically significant at the 24 h time point. Second, the decentralized preparations are typically run side by side with control preparations and thus kept in nearly identical conditions. They would therefore be expected to be subject to the same level of run down due to any uncontrolled external conditions as the control preparations. Yet, decentralized preparations show a clear decrease in variance, achieving values indistinguishable from control by 24 h. We interpret this to mean that the recovery of the robustness of the decentralized preparations is stronger than its run down despite the general deterioration (or run down) of the preparations *in vitro*.

**TABLE 4:** Control pyloric rhythm variances across a 9–30°C temperature range.

	Time (hours)	Control variances	CI (low)	CI (high)
PD_off_	0	0.00056	0.00089	0.00040
	0.5	0.00040	0.00088	0.00027
	6	0.00103^a^	0.00169	0.00074
	24	0.00123^a^	0.00202	0.00079
LP_on_	0	0.00100	0.00160	0.00071
	0.5	0.00086	0.00187	0.00057
	6	0.00186	0.00307	0.00134
	24	0.00207	00.372	0.00144
LP_off_	0	0.00523	0.00814	0.00385
	0.5	0.00431	0.00855	0.00288
	6	0.00694	0.01092	0.00508
	24	0.00751	0.01273	0.00533

Pyloric rhythm phases PD_off_, LP_on_, and LP_off_ were compared across time in control preparations as part of the Generalized Linear Model (GLM) test reported in Table 2. Time-dependent comparisons: ^a^*P* < 0.05 between 0.5 h and the indicated times. CI, confidence interval. Df = 160.

In this analysis of robustness, we have included only the phase relationships within the pyloric network and not the network oscillatory frequency. It is widely recognized that these phase relationships are extremely stable while the frequency can vary several fold (Marder and Bucher, 2007). Pyloric activity drives the rhythmic movements of muscles involved in what is believed to be the filtering activity of the semi-digested food passing through the pyloric chamber (McGaw and Curtis, 2013). This occurs by a peristaltic movement of food through a series of structures that can trap particles according to their size. Thus, while the frequency of the network can be allowed to vary (when food is abundant, for example), the phase relationships typically need to remain constant to ensure the correct muscle contraction sequences that enable that the movement of food particles proceeds in an orderly fashion.

Is variance an appropriate measure of robustness? Various measures of robustness have been proposed [e.g., (Tang et al., 2012; Revilla-Vallejo et al., 2023)], or it is often only qualitatively assessed. We chose the simplest possible measure that will capture what we consider the essence of a robust system: the ability of the system to withstand perturbations without significant deviation from control levels of the features that define the activity of the system. This ability is, in its simplest form, captured by the variance of the means of phase relationships measured across a range of perturbations, in this case, a range of temperatures.

### Mechanisms of robustness recovery

The reduction in robustness upon removal of neuromodulatory input was already observed and reported earlier for both the pyloric network activity (Haddad and Marder, 2018), as well as that of a second functional network found in the stomatogastric ganglion, the gastric mill network activity (Stadele and Stein, 2022). In the case of the gastric mill activity, robustness appears to be maintained by a neuromodulator-dependent mechanism that involves neuromodulator-containing neurons that release their content(s) in a temperature-dependent fashion (Stadele et al., 2015). According to this, it appears that increased temperature in the absence of neuromodulators leads to an increase in leak conductance of the network neurons (Stadele et al., 2015). However, an increase in temperature in the presence of at least one neuromodulator (*Cancer borealis* tachykinin-related peptide Ia, CabTRP Ia) enhanced release at higher temperatures and induced an active reduction in leak conductance via a mechanism previously described in pyloric network neurons: the effective enhancement of a negative leak conductance (Zhao et al., 2010). It is not yet known what mechanisms might stabilize activity or its robustness in the pyloric network. A gradual reduction of leak conductance after decentralization is a possibility that should be tested.

On the other hand, phase constancy has been shown to be dependent on synaptic depression and intrinsic current interactions, in particular, that of a transient K^+^ current, I_*A*_ (Manor et al., 2003; Greenberg and Manor, 2005) and I_*H*_ (Harris-Warrick et al., 1995). More recently, phase constancy at different temperatures has been shown to be generated by the non-linear combination of several voltage and temperature-dependent currents (Caplan et al., 2014; Alonso and Marder, 2020). It is known that at least in one pyloric network cell type, PD neurons, I_*A*_ decreases 24 h after decentralization (Khorkova and Golowasch, 2007). A reduction in I_*A*_ would be expected to phase advance the bursting of a neuron, which is what we observed after decentralization. However, I_*A*_ only recovers to control values in PD neurons of decentralized preparations after ∼5 days (Khorkova and Golowasch, 2007) and could thus not fully explain our observations. However, simultaneous changes of I_*A*_ and synaptic currents in the appropriate combination could account for our results. More broadly, it is likely that the entire conductance space of each neuron (the set of all ionic conductances expressed by each cell) completely reorganizes in such a way as to allow the restoration of the pyloric activity and its robustness by a different combination of intrinsic and synaptic conductances. The pyloric network, and many other systems, are well known for their degeneracy, meaning their ability to generate similar activity by a large number of different conductance combinations (Drion et al., 2015; Prinz, 2017; Calabrese, 2021; Goaillard and Marder, 2021), and we know that such reorganization does take place (Khorkova and Golowasch, 2007; Ransdell et al., 2012; Temporal et al., 2012). Activity-dependent mechanisms are known to regulate conductance levels and relationships (Turrigiano et al., 1994; Golowasch et al., 1999a), and they could also possibly drive the recovery of phase relationships (O’Leary and Marder, 2016). Alonso and Marder (2020) have shown that phase constancy across temperatures can be generated in pyloric network models by multiple combinations of conductances. In this network, the absence of neuromodulatory activity means the deactivation of key modulator-activated ionic conductances involved in rhythm generation (Bose et al., 2014; Schneider et al., 2021). This absence may also be a signal that relaxes constraints on conductance space across the pyloric network, allowing these conductances to homeostatically drift in this space to a new state in which resilient activity and functionally appropriate phase relationships are generated (Golowasch, 2019).

In the mammalian respiratory network, the density of pacemakers seems to be an important factor that promotes robustness (Purvis et al., 2007). In the pyloric network, there is only one pacemaker (the Anterior Burster, AB, neuron) and five additional neuronal types (for a total of 11 neurons). Two additional neurons (Pyloric Dilators, PD, neurons) form part of what is called the pacemaker kernel (Marder and Bucher, 2007; Figure 1) and may express pacemaker properties, at least under certain conditions, such as when the network is decentralized (Thoby-Brisson and Simmers, 1998, 2000; Luther et al., 2003). Thus, the recovery of the robustness of the pyloric network after decentralization may, in part, also be due to the increase in the number of effective pacemaker neurons.

In conclusion, we show that the robustness of pyloric network activity appears to be a feature linked to the ability of the network to produce stable rhythmic activity since both seem to recover in tandem after a major disruption, i.e., decentralization, which changes not just neuromodulatory input to the network neurons but also disrupts their activity. The mechanism leading to this recovery is not known, but it is likely to involve simultaneous changes at multiple levels of conductance expression: synaptic as well as intrinsic, perhaps driven by similar activity-dependent mechanisms. This recovery of activity and robustness may be a feature shared by other networks and species where the absence of neuromodulators may aid in this recovery after an insult or disease.

## Data availability statement

The raw data supporting the conclusions of this article will be made available by the authors, without undue reservation.

## Ethics statement

The manuscript presents research on animals that do not require Ethical Approval for their study.

## Author contributions

JG: Conceptualization, Funding acquisition, Investigation, Methodology, Project administration, Writing – review and editing. SM-P: Conceptualization, Data curation, Formal analysis, Investigation, Methodology, Software, Writing – original draft, Writing – review and editing.

## Footnotes

1
https://www.ncei.noaa.gov/access/coastal-water-temperature-guide/natl.html
